# ACSL3–PAI-1 signaling axis mediates tumor-stroma cross-talk promoting pancreatic cancer progression

**DOI:** 10.1126/sciadv.abb9200

**Published:** 2020-10-30

**Authors:** Matteo Rossi Sebastiano, Chiara Pozzato, Maria Saliakoura, Zhang Yang, Ren-Wang Peng, Mirco Galiè, Kevin Oberson, Hans-Uwe Simon, Evanthia Karamitopoulou, Georgia Konstantinidou

**Affiliations:** 1 Institute of Pharmacology, University of Bern, 3010 Bern, Switzerland.; 2 Division of General Thoracic Surgery, Inselspital, Bern University Hospital, 3008 Bern, Switzerland.; 3 Department of Neuroscience, Biomedicine and Movement, University of Verona, 37134 Verona, Italy.; 4 Department of Clinical Immunology and Allergology, Sechenov University, Moscow, Russia.; 5 Institute of Pathology, University of Bern, 3008 Bern, Switzerland.

## Abstract

Pancreatic ductal adenocarcinoma (PDAC) is characterized by marked fibrosis and low immunogenicity, features that are linked to treatment resistance and poor clinical outcomes. Therefore, understanding how PDAC regulates the desmoplastic and immune stromal components is of great clinical importance. We found that acyl-CoA synthetase long-chain 3 (ACSL3) is up-regulated in PDAC and correlates with increased fibrosis. Our in vivo results show that *Acsl3* knockout hinders PDAC progression, markedly reduces tumor fibrosis and tumor-infiltrating immunosuppressive cells, and increases cytotoxic T cell infiltration. This effect is, at least in part, due to decreased plasminogen activator inhibitor–1 (PAI-1) secretion from tumor cells. Accordingly, *PAI-1* expression in PDAC positively correlates with markers of fibrosis and immunosuppression and predicts poor patient survival. We found that PAI-1 pharmacological inhibition strongly enhances chemo- and immunotherapeutic response against PDAC, increasing survival of mice. Thus, our results unveil ACSL3–PAI-1 signaling as a requirement for PDAC progression with druggable attributes.

## Teaser

Pharmacological inhibition of PAI-1 strongly enhances chemo- and immunotherapeutic response against PDAC.

## INTRODUCTION

Pancreatic ductal adenocarcinoma (PDAC) represents 95% of all pancreatic cancers and, with a 5-year survival rate of only 7%, is one of the deadliest malignancies (*1*). Surgery remains the only yet insufficient approach for the treatment of PDAC, as this carcinoma is highly refractory to all available antitumor pharmacological options (*2*). This is, at least in part, due to the strong desmoplastic reaction associated with PDAC progression, displaying a strong activation of pancreatic stellate cells (PSCs) and formation of dense extracellular matrix that causes insufficient tumor perfusion and acts as an impenetrable barrier to intravenously injected anticancer molecules or chemotherapeutic agents (*3*). Moreover, tumor-associated macrophages (TAMs) including M2-like macrophages (or alternatively activated macrophages) promote fibrosis and inhibit both adaptive and innate antitumor immunity by secreting immunosuppressive molecules including transforming growth factor–β (TGF-β), interleukin-10 (IL-10), and arginase 1 (Arg1), thus negatively affecting patient outcomes (*4*, *5*). Therefore, a better understanding of the biology of PDAC, including the mechanisms linking its progression with the regulation of its stromal components, is urgent, as it will provide insights that can be translated into needful therapeutic strategies.

The most common gene alterations in human PDACs include mutations in the *KRAS* oncogene (95% of cases) and inactivating mutations in the *TP53* tumor suppressor (75% of cases) (*6*). Mouse models have recapitulated this observation, where *Kras* activation in combination with a point mutation or deletion of one copy of the *Trp53* gene is sufficient to induce PDAC with characteristic features of the human disease, including progression from preinvasive pancreatic intraepithelial neoplasia lesions (PanINs) to invasive PDACs and strong association with desmoplastic stroma (*7*–*9*). Hence, these genetically engineered mouse models provide a unique opportunity to interrogate the functional molecular requirements for PDAC progression and to define features such as the desmoplastic stromal reaction.

We previously found that the acyl–coenzyme A (CoA) synthetase long-chain family member 3 (ACSL3) is necessary for the extracellular lipid utilization and proliferation of KRAS-driven cancer cells because of its role in activation of extracellularly derived long-chain fatty acids (*10*). The proliferation of PDAC strongly depends on extracellularly derived fatty acids, including lipids derived from stromal components, such as PSCs (*11*, *12*). Thus, we hypothesized that ACSL3 is a crucial requirement for this malignancy and that studying the functional effect of ACSL3 suppression will unmask important requirements of PDAC that could be therapeutically exploited. Here, by using genetically defined *Kras^G12D^*-driven pancreatic cancer mouse models, human pancreatic ductal epithelial (HPDE) cell lines, human patient samples, and epidemiological data, we interrogated the role of ACSL3 signaling in PDAC progression to uncover an underlying tumorigenic mechanism with druggable attributes.

## RESULTS

### ACSL3 is up-regulated in human PDAC

**Fig. 1:**
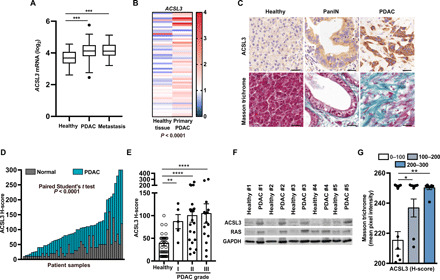
ACSL3 is overexpressed in human PDAC. (**A**) mRNA levels of *ACSL3* in patient healthy tissue, primary PDAC, and PDAC metastasis from the subset GSE71729. Healthy pancreatic tissue: *n* = 46 patients, primary PDAC: *n* = 145 patients, and PDAC metastasis: *n* = 61 patients. Data are represented as box (Whisker’s) plot. Dots evidence outliers according to Tukey’s method. (**B**) mRNA levels of *ACSL3* in patient primary PDAC and matched adjacent healthy tissue from the subset GSE62452 (*n* = 60). Data are expressed as entire numbers normalized by global average. (**C**) Representative IHC staining for ACSL3 (top) and extracellular matrix deposition marker with Masson trichrome (blue, bottom) of a human TMA showing primary healthy tissue, PanIN, and primary PDAC. Scale bars, 50 μm. (**D**) ACSL3 IHC quantification (H-score) of TMA comparing PDAC and adjacent healthy tissue (*n* = 50 samples). We considered an H-score of less than 100, from 100 to 200, and above 201 having a low, intermediate, or high staining intensity, respectively. (**E**) Stratification of the patients by tumor grade from (D). Healthy tissue, *n* = 50 samples; grade I, *n* = 5 samples; grade II, *n* = 23 samples; grade III, *n* = 22 samples. (**F**) Immunoblot for ACSL3, total RAS, and glyceraldehyde-3-phosphate dehydrogenase (GAPDH) of five human patient-derived tumor samples and matched adjacent healthy tissue. (**G**) Correlation between ACSL3 and Masson trichrome staining of TMA from (C) and (D). Samples are divided in low, intermediate, and high ACSL3 staining. We considered an H-score of less than 100, from 100 to 200, and above 201 having a low, intermediate, or high staining intensity, respectively. Error bars represent mean ± SD; statistical analysis was performed using one-way ANOVA. *P < 0.05, **P < 0.01, ***P < 0.001, and ****P < 0.0001.

To assess the expression level of *ACSL3* in human pancreatic cancer, we examined publicly available human patient-derived data from the Gene Expression Omnibus (GEO) database (subset GSE71729). Our analysis evidenced that the mRNA level of *ACSL3* is higher in primary ductal adenocarcinomas and metastasis compared to healthy epithelium (Fig. 1A). These results were additionally confirmed with paired analysis from matched healthy and PDAC human samples (dataset GSE62452), showing a marked increase in the expression of *ACSL3* compared to adjacent healthy tissue from the same patients (Fig. 1B).

Next, we performed immunohistochemistry (IHC) for ACSL3 on a tissue microarray (TMA) containing 50 PDAC patient samples. We scored for low, intermediate, or high ACSL3 protein levels based on a histological score that takes into consideration the percentage of cells stained at different intensities (H-score). We found that ACSL3 was barely detectable in the normal pancreatic epithelium, modestly expressed in PanINs, and markedly elevated in PDAC (Fig. 1, C and D, and fig. S1A). Notably, we found that a high ACSL3 H-score positively correlates with a higher tumor histological grade (Fig. 1E). Next, to confirm the above results, we used matched healthy and PDAC tissue from five pancreatic cancer patients, and we assessed ACSL3 protein levels by immunoblot analysis. This analysis evidenced a consistent increase of ACSL3 protein in PDAC compared to healthy tissue (Fig. 1F).

To determine whether high levels of ACSL3 in PDAC cells correlate with elevated stromal fibrosis, we stratified human PDAC patient samples on the basis of low, intermediate, or high ACSL3 H-score and assessed the degree of collagen deposition by Masson trichrome staining. Notably, this analysis evidenced that high ACSL3 protein levels are associated with a higher degree of fibrosis (Fig. 1, C and G, and fig. S1B). Together, the data strongly suggest a positive role of ACSL3 in PDAC progression and desmoplastic stromal reaction.

### *Acsl3* deletion delays PDAC progression and reduces fibrosis in mice

**Fig. 2:**
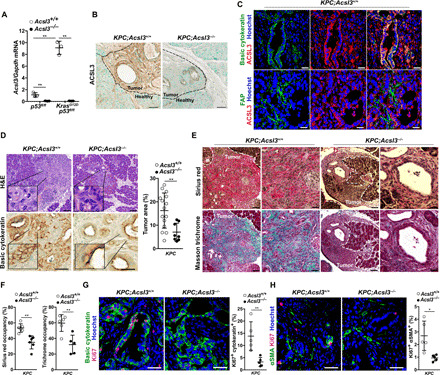
*Acsl3* knockout reduces tumor fibrosis and suppresses tumor progression. (**A**) *Acsl3* mRNA levels from healthy tissue (*Kras^+/+^*;*p53^flox/flox^*) either *Acsl3^+/+^* or *Acsl3^−/−^* and dissected tumors from *LSL-Kras^G12D/+^*;*p53^flox/flox^*;*Pdx1-Cre^ERT2^*;*Acsl3*^+/+^ (*KPC*;*Acsl3^+/+^*) and *Kras^G12D/+^*;*p53^flox/flox^*;*Pdx1-Cre^ERT2^*;*Acsl3^−/−^* (*KPC*;*Acsl3^−/−^*) (*n* = 3 mice per group). (**B**) Representative IHC staining images of ACSL3 from *KPC*;*Acsl3^+/+^* and *KPC*;*Acsl3^−/−^* pancreas sections representing healthy and tumor tissue. Scale bars, 50 μm. (**C**) Representative images of immunofluorescent staining for basic cytokeratin (top, green), ACSL3 (red), nuclei (blue), and FAP (bottom, green) of *KPC*;*Acsl3^+/+^* tumor-bearing pancreas sections. Scale bars, 70 μm. (**D**) Representative images of H&E-stained (top, left) and basic cytokeratin–stained (bottom, left) tumor-bearing pancreas sections and pancreas tumor area quantification (right) reported as percentage of tumor area per total pancreas area from H&E-stained mouse pancreas sections of *KPC*;*Acsl3^+/+^* (*n* = 15) and *KPC*;*Acsl3^−/−^* (*n* = 8) mice. Scale bars, 70 μm (top) and 50 μm (bottom). (**E**) Representative images of Sirius red–stained (top) and Masson trichrome–stained (bottom) mouse pancreatic cancer sections of *KPC*;*Acsl3^+/+^* and *KPC*;*Acsl3^−/−^* mice. Scale bars, 70 μm (left) and 30 μm (right). (**F**) Quantification of fibrosis based on Sirius red and Masson trichrome staining, respectively, reported as % of area occupancy over tumor area assessed on a representative pancreas section per mouse (*n* = 5 to 6 mice per group). (**G**) Representative immunofluorescence staining for basic cytokeratin, Ki67, and nuclei (Hoechst) (left) and relative quantification (right) of *KPC*;*Acsl3^+/+^* and *KPC*;*Acsl3^−/−^* tumor-bearing pancreas sections. The quantifications are the average of 15 pictures per mouse (*n* = 5 mice per group). Scale bars, 50 μm. (**H**) Representative immunofluorescence staining for αSMA, Ki67, and nuclei (Hoechst) (left) and relative quantification (right) of *KPC*;*Acsl3^+/+^* and *KPC*;*Acsl3^−/−^* pancreatic cancer tissue sections. The quantifications are the average of 15 pictures per mouse; *n* = 5 mice per group. Scale bars, 50 μm. Error bars represent mean ± SD; statistical analysis was performed using Student’s *t* test or one-way ANOVA. *P < 0.05 and **P < 0.01.

To assess the impact of *Acsl3* deletion on PDAC progression in a genetically defined mouse model, we generated mice carrying a transgene, allowing tamoxifen-dependent expression of the *Cre* recombinase under the pancreas-specific *Pdx-1* promoter (*Pdx1-Cre^ERT2^*), a *Cre*-activatable *Kras^G12D^* allele (*LSL-Kras^G12D/+^*), homozygous for a *Cre-*conditional *p53* knockout allele (*p53^floxflox^*), and either wild type or homozygous for an *Acsl3* knockout allele (*Acsl3*^+/+^ or *Acsl3*^−/−^, respectively), to generate two experimental groups: *LSL-Kras^G12D/+^*;*p53^flox/flox^*;*Pdx1-Cre^ERT2^*;*Acsl3*^+/+^ and *LSL-Kras^G12D/+^*; *p53^flox/flox^*;*Pdx1-Cre^ERT2^*;*Acsl3*^−/−^ (hereafter named *KPC*;*Acsl3*^+/+^ and *KPC*;*Acsl3*^−/−^, respectively). Tamoxifen injection to these mice drives the *Cre*-mediated recombination and results in the excision of the *loxP*-flanked stop codon (LSL), thereby leading to conditional expression of *Kras^G12D^* and deletion of *p53* specifically in the mouse pancreas (fig. S2A) (*13*, *14*). The *KPC*;*Acsl3^−/−^* mice were born according to the expected Mendelian ratio and without obvious macroscopic defects during development or adult life compared to the *KPC*;*Acsl3^+/+^* mice. We assessed tumor formation at 8 weeks after tamoxifen-mediated induction, a time point where the pancreas is occupied by low- and high-grade pancreatic cancer. The analysis of pancreata from *KPC*;*Acsl3*^−/−^ mice evidenced a complete loss of *Acsl3* mRNA, protein, and a reduction in total ACSL activity compared to the *KPC*;*Acsl3*^+/+^ healthy and tumor tissue, confirming the knockout of *Acsl3* (Fig. 2, A and B, and fig. S2B). In agreement with the human data (Fig. 1), tumor tissue of the *KPC*;*Acsl3*^+/+^ mice showed a marked increase in *Acsl3* mRNA and total ACSL activity compared to healthy tissue (Fig. 2A and fig. S2B). Furthermore, results from our IHC analysis showed that, in the pancreata of these mice, ACSL3 is present in tumors, but not in the healthy surrounding tissue (Fig. 2B). We also found that within the tumor lesions, ACSL3 is broadly expressed in tumor cells and only to a limited extent in stromal fibroblasts as evidenced by costaining with basic cytokeratin, a marker of epithelial cells (colocalization score, 68.23 ± 7.14%) and fibroblast activation protein α (FAP; colocalization score, 3.63 ± 2.18%), respectively (Fig. 2C).

Histological evaluation of hematoxylin and eosin (H&E)–stained tissue sections revealed decreased disease progression in *KPC*;*Acsl3*^−/−^ compared to *KPC*;*Acsl3*^+/+^ mice (Fig. 2D). Moreover, the pancreas weight of *KPC*;*Acsl3*^−/−^ mice was slightly reduced compared to *KPC*;*Acsl3*^+/+^ (fig. S2C). The fibrotic tissue measured by both Masson trichrome and Sirius red staining was decreased in *KPC*;*Acsl3*^−/−^ compared to *KPC*;*Acsl3*^+/+^ tumors (Fig. 2, E and F). This degree of reduction in desmoplastic tissue was further confirmed by a reduction in the myofibroblast markers α–smooth muscle actin (αSMA) and FAP (fig. S2D).

To better understand the mechanism leading to decreased tumor formation upon *Acsl3* deletion, we evaluated tumor cell proliferation by Ki67 staining. We found that *Acsl3* deletion reduced the percentage of Ki67^+^ cells in both tumor cells (Ki67^+^/cytokeratin^+^) and tumor stromal fibroblasts (Ki67^+^/αSMA^+^) (Fig. 2, G and H). Notably, we did not detect an increase in cell death as evaluated by TUNEL (terminal deoxynucleotidyl transferase–mediated deoxyuridine triphosphate nick end labeling) assay (fig. S2E). Together, these data suggest that ACSL3 supports the progression to PDAC by enhancing both tumor and stromal cell proliferation.

The postnatal KPC model used for this study is highly relevant, as human PDAC is an adult-onset malignancy. However, in this model, *Pdx1*-*Cre*–mediated recombination is observed only in the islet and acinar cells (*15*), raising the possibility of a context-specific phenotype. To address this issue, we tested the impact of *Acsl3* knockout in a prenatal mouse model of PDAC in which Cre is expressed in almost all pancreatic cell types. We generated mice carrying the *LSL-Kras^G12D/+^* allele, homozygous for the *p53^floxflox^* allele, either *Acsl3*^+/+^ or *Acsl3*^−/−^, and bearing a transgene allowing a constitutive expression of the Cre recombinase under the pancreas-specific *Pdx1* (*Pdx1-Cre^6tuv^*) promoter to generate two experimental groups: *LSL-Kras^G12D/+^*;*p53^flox/flox^*;*Pdx1-Cre^6tuv^*;*Acsl3*^+/+^ and *LSL-Kras^G12D/+^*;*p53^flox/flox^*;*Pdx1-Cre^6tuv^*;*Acsl3*^−/−^ (hereafter *KPCC*;*Acsl3*^+/+^ and *KPCC*;*Acsl3*^−/−^, respectively) (fig. S2F). The *Pdx1-Cre^6tuv^* allele allows the expression of *Cre* recombinase under the pancreas-specific *Pdx1* promoter starting at approximately embryonic day 8.5. At this time, the *Pdx1* gene is expressed in pre-pancreatic endoderm, thus allowing *Cre* expression (hence tumor initiation) in virtually all pancreatic cell types (*16*–*18*). We sacrificed the mice at 6 weeks of age, a time point where the mouse pancreas is highly occupied by high-grade PDAC lesions. In agreement with our results from the inducible *KPC* model, pancreas weight, tumor area, and fibrotic tissue were markedly decreased in *KPCC*;*Acsl3^−/−^* compared to *KPCC*;*Acsl3^+/+^* mice (fig. S2, G to I).

### *Acsl3* deletion blocks PDAC immunosuppression in mice

Preinvasive and invasive PDAC lesions from mouse models and human patients are characterized by a strong presence of immunosuppressive cell types including tumor-associated M2-like macrophages, myeloid-derived suppressor cells, and regulatory T cells (T_regs_) with a concomitant lack of effector T cells (*18*–*22*). Notably, a growing amount of evidence indicates that tumor-associated fibrosis promotes PDAC progression partly by negatively regulating antitumor immunity (*23*). Therefore, we investigated whether the reduced PDAC progression and fibrosis, upon *Acsl3* knockout, was associated with changes in tumor immune cell infiltration.

**Fig. 3:**
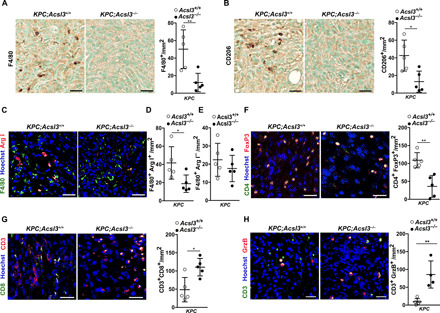
ACSL3 drives tumor microenvironment toward immunosuppression. (**A** and **B**) Representative images of IHC staining (left) and quantification (right) of F4/80 and CD206, respectively, in PDAC lesions of *KPC*;*Acsl3^+/+^* and *KPC*;*Acsl3^−/−^* tumor-bearing pancreas sections; *n* = 5 mice per group. Scale bars, 50 μm. (**C**) Representative images of immunofluorescence costaining of the pan-macrophage marker F4/80 with the M2 macrophage marker Arg1 of *KPC*;*Acsl3^+/+^* and *KPC*;*Acsl3^−/−^* tumor-bearing pancreas sections; *n* = 5 mice per group. Scale bars, 30 μm. (**D** and **E**) Quantifications from (C) expressed as double-positive (F4/80^+^/Arg1^+^) and single-positive (F4/80^+^/Arg1^−^) cells per mm^2^, respectively. The quantifications are the average of 15 pictures per mouse; *n* = 5 mice per group. (**F**) Representative images of immunofluorescence staining (left) and quantification (right) of CD4 (green), FoxP3 (red), and nuclei (blue) of *KPC*;*Acsl3^+/+^* and *KPC*;*Acsl3^−/−^* tumor-bearing pancreas sections. The quantifications are the average of 15 pictures per mouse; *n* = 5 mice per group. Scale bars, 50 μm. (**G**) Representative images of immunofluorescence staining (left) and quantification (right) of CD3 and CD8, reported as positive cells per mm^2^ of *KPC*;*Acsl3^+/+^* and *KPC*;*Acsl3^−/−^* tumor-bearing pancreas sections; *n* = 5 mice per group. Scale bars, 50 μm. (**H**) Representative images of immunofluorescence staining (left) and quantification (right) of CD3 and granzyme B (GrzB) reported as positive cells per mm^2^ of *KPC*;*Acsl3^+/+^* (*n* = 5 mice per group) and *KPC*;*Acsl3^−/−^* (*n* = 4 mice per group) tumor-bearing pancreas sections. Scale bars, 50 μm. Error bars represent mean ± SD; statistical analysis was performed using unpaired Student’s *t* test. *P < 0.05 and **P < 0.01.

First, we revealed a strong reduction of TAM infiltration in tumors of *KPC*;*Acsl3^−/−^* mice compared to *KPC*;*Acsl3^+/+^* mice as shown by staining with the macrophage markers F4/80 and CD206 (Fig. 3, A and B). Strikingly, we found that the number of alternatively activated macrophages (M2) expressing Arg1 was reduced in *KPC*;*Acsl3^−/−^* pancreata compared to *KPC*;*Acsl3^+/+^* (Fig. 3, C and D). The number of macrophages negative for Arg1 (F4/80^+^/Arg1^−^) was unaltered, suggesting that the macrophage decrease in *KPC*;*Acsl3^−/−^* pancreata was due to a decrease in the M2 macrophage population (Fig. 3E). Neutrophils have been recently identified as potential contributors to mutant Kras-induced lung cancer progression (*24*, *25*). Thus, we assessed neutrophil abundance by Ly6g staining in pancreatic cancer tissue sections from *KPC*;*Acsl3^+/+^* and *KPC*; *Acsl3^−/−^* mice, but we did not find any difference in neutrophil numbers (fig. S3A).

Next, we assessed the presence of T_regs_ by costaining with the surface marker CD4 and the nuclear transcription factor FoxP3 and found that the *KPC*;*Acsl3^−/−^* tumors contained 50% less T_regs_ compared to *KPC*;*Acsl3^+/+^* tumors (Fig. 3F). Moreover, immunofluorescence analysis of CD3^+^/CD8^+^ cytotoxic T lymphocytes (CTLs) revealed a twofold increase in the number of CD8^+^ T cells in *KPC*;*Acsl3^−/−^* mice with a concomitant increase in granzyme B expression, indicative of functional CTLs (Fig. 3, G and H). Notably, the effect of *Acsl3* loss on the different immune cell populations was tumor specific; as in the healthy *Pdx1-Cre^ERT2^*;*p53^flfl^*;*Acsl3^+/+^* and *Pdx1-Cre^ERT2^*; *p53^flfl^*; *Acsl3^−/−^* mice, we did not detect differences in the total number of T cells (CD45^+^/CD3^+^), CD4^+^ T cells (CD45^+^/CD3^+^/CD4^+^), or CD8^+^ T cells (CD45^+^/CD3^+^/CD8^+^) (fig. S3B).

The decreased tumor immunosuppression upon *Acsl3* deletion was also recapitulated in *KPCC*;*Acsl3^−/−^* compared to *KPCC*;*Acsl3^+/+^* mice. Also in this context, we found that *Acsl3* deletion leads to a strong decrease in the intratumoral number of M2-polarized macrophages (F4/80^+^/Arg1^+^) and T_regs_ (CD4^+^/FoxP3^+^) and a significant increase in the number of CTLs (CD3^+^/CD8^+^) (fig. S3, C to E). Together, our results in PDAC mouse models strongly suggest that the loss of ACSL3 lowers immunosuppression and improves the antitumor CTL response.

### ACSL3 promotes the expression of PAI-1 in PDAC

**Fig. 4:**
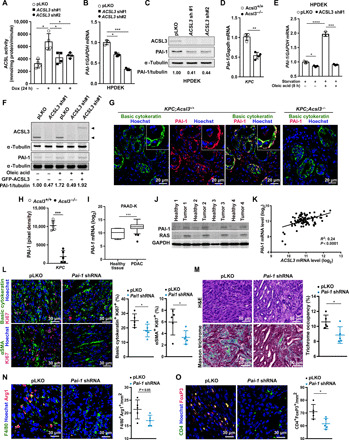
ACSL3 controls PAI-1 levels in PDAC. (**A**) ACSL activity of HPDE cells transduced with a pLKO empty vector or shRNAs against *ACSL3* and treated as indicated; *n* = 4 per group. (**B** and **C**) *PAI-1* mRNA levels (B) and immunoblot (C) of HPDEK cells transduced as in (A). (**D**) *Pai-1* mRNA levels from tumors of *KPC*;*Acsl3^+/+^* and *KPC*;*Acsl3^−/−^* mice; *n* = 4 mice per group. (**E** and **F**) *PAI-1* mRNA levels (E) and immunoblot (F) of HPDEK cells treated as indicated; *n* = 3. A pEGFP-C1-ACSL3_HA_ plasmid was used to rescue ACSL3 upon knockdown. (**G** and **H**) Representative immunofluorescence staining images of basic cytokeratin, PAI-1, and Hoechst (nuclei) (G) and PAI-1 quantification (H) of *KPC*;*Acsl3^+/+^* and *KPC*;*Acsl3^−/−^* tumor tissue sections; *n* = 5 mice per group. (**I**) *PAI-1* mRNA levels from human healthy tissue (*n* = 4) and primary PDAC (*n* = 124) from subset PAAD-K (KRAS mutant). In the Whisker’s plot, dots represent 5 to 95 percentile. (**J**) Immunoblot for PAI-1 and RAS of four human patient-derived matched healthy per tumor tissue. (**K**) Correlation between *ACSL3* and *PAI-1* mRNA levels in PDAC human samples (subset PAAD-K); *n* = 94. (**L** to **O**) Representative immunofluorescence staining images (left) and quantifications (right) of the indicated targets from pancreas sections of KP orthotopic PDAC tumors with or without *Pai-1* knockdown; *n* = 5 mice per group. The quantifications are the average of 15 pictures per mouse. Error bars represent mean ± SD; statistical analysis was performed using unpaired Student’s *t* test or one-way ANOVA. *P < 0.05, **P < 0.01, ***P < 0.001, and ****P < 0.0001.

Our results show that *Acsl3* is mainly expressed in tumor cells (Fig. 2C), suggesting that the reduction of tumor fibrosis and immunosuppression in *Acsl3* knockout mice is specifically due to *Acsl3* deletion in tumor cells. Thus, we hypothesized that *Acsl3* deletion could interfere with the cross-talk between tumor and stromal cells by affecting the secretion of soluble factors from tumors such as cytokines. To test this hypothesis, we used a healthy human pancreatic ductal epithelial cell line (HPDE), which harbors a doxycycline (Dox)–inducible oncogenic *KRAS^G12D^* expression (thereafter named HPDEK) (*26*). In these cells, Dox treatment for 24 hours induced *KRAS^G12D^* expression and increased the ACSL3 transcription, protein level, and the total ACSL activity (Fig. 4A and fig. S4, A and B). Notably, the increased ACSL activity was completely abolished by *ACSL3* knockdown (Fig. 4A). Next, we transduced the HPDEK cells with either an empty vector control or a short hairpin RNA (shRNA) against *ACSL3* and measured the relative levels of 36 cytokines in the culture supernatant using a human cytokine array. The only significant difference identified by this analysis was a reduction in plasminogen activator inhibitor type 1 (PAI-1) upon *ACSL3* knockdown (fig. S4, C and D). The PAI-1 decrease was confirmed by quantitative real-time polymerase chain reaction (qPCR) and immunoblot with a second shRNA against *ACSL3* (Fig. 4, B and C). Moreover, qPCR from mouse tumor samples evidenced a decrease in *Pai-1* mRNA in *KPC*;*Acsl3*^−/−^ tumors compared to *KPC*;*Acsl3^+/+^* tumors, additionally confirming our in vitro results (Fig. 4D). Last, *KRAS^G12D^* induction with Dox increased the transcriptional and protein level of PAI-1 in HPDE cells (fig. S4, E and F).

PAI-1 is a profibrotic TGF-β–responsive gene and has been shown to be positively regulated by long-chain unsaturated fatty acids including oleic and linoleic acid (*27*). To assess causality between ACSL3 and PAI-1 levels, we supplied cells with oleic acid, which is also a lipid substrate of ACSL3, to lipid-starved HPDEK cells with or without *ACSL3* knockdown. qPCR and immunoblot analysis evidenced that oleic acid increased the transcriptional and protein levels of PAI-1, and this was abolished by *ACSL3* knockdown, additionally confirming that ACSL3 is necessary for *PAI-1* induction (Fig. 4, E and F).

In *KPC*;*Acsl3*^+/+^ pancreatic tissue, PAI-1 was mainly localized in cancer cells (Fig. 4G; colocalization score PAI-1/basic cytokeratin: 78.27 ± 4.11%) and appeared enriched at their periphery and in close contact with the adjacent stromal cells (Fig. 4G, insets). Moreover, PAI-1 only minimally colocalized with both fibroblast markers FAP and αSMA in *KPC*;*Acsl3^+/+^* tumors (colocalization PAI-1/FAP: 7.61 ± 0.87% and PAI-1/αSMA: 5.23 ± 1.62%; fig. S4G). Markedly, PAI-1 was significantly decreased in *Acsl3^−/−^* tumors compared to *Acsl3^+/+^* tumors (Fig. 4H).

To evaluate whether *PAI-1* and *ACSL3* expression levels are positively correlated in human patient-derived PDAC, we performed an analysis on publicly available RNA-sequencing data from The Cancer Genome Atlas (TCGA; subset PAAD) (*28*). For this analysis, we took into consideration only *KRAS*-mutated samples (thereafter named PAAD-K). PDAC samples evidenced a significantly higher *PAI-1* expression levels compared to healthy pancreatic tissue (Fig. 4I). Furthermore, immunoblot analysis of PAI-1 in matched healthy and tumor patient-derived tissue revealed a marked increase in PAI-1 protein level in tumor tissue (Fig. 4J). Further analysis evidenced a positive correlation between the expression levels of *PAI-1* and *ACSL3*, suggesting a co-regulation in PDAC (Fig. 4K).

### Tumor-derived PAI-1 supports PDAC progression

PAI-1 belongs to the superfamily of serine-protease inhibitors, which, by inhibiting the urokinase-type plasminogen activator and plasmin-dependent matrix metalloproteinases, promotes fibrosis (*29*, *30*). Furthermore, recent evidence suggested that PAI-1 promotes M2 macrophage polarization, thus stimulating immunosuppression (*31*). Our findings indicated that PAI-1 is produced and secreted by pancreatic cancer cells in vitro (fig. S4D), and it is enriched in the outer edge of cancer cells, next to stromal components (Fig. 4G and fig. S4G). Moreover, the *KPC*;*Acsl3*^−/−^ tumors displayed reduced fibrosis and immunosuppression (Figs. 2 and 3). Thus, our data suggest that PAI-1 may be a key intermediate of tumor/stroma cross-talk, mediating immunosuppression and fibrosis in PDAC. To assess the impact of tumor-derived PAI-1 on tumor/stroma interaction, we used a cancer cell line derived from a mouse pancreatic cancer model, *Kras^G12D^*;*p53^R172H^* (thereafter named KP cells) (*8*). Orthotopic injection of these cells in the pancreata of immunocompetent mice generates a spontaneous stromal response and displays common patterns with disease progression (*32*). To confirm that the KP cells are relevant concerning PAI-1 regulation, we transduced cells with an shRNA against *Acsl3* or an empty vector control and performed a mouse cytokine array comprising 111 cytokines. We found that PAI-1 was one of the most significantly decreased cytokines following *Acsl3* knockdown in KP cells (fig. S4H). Moreover, this analysis revealed a decrease in the level of additional proteins associated with collagen deposition and remodeling including collagen type XVIII alpha 1 (Col18a1), insulin-like growth factor binding protein 3 (IGFBP3), and matrix metalloproteinase 3 (MMP3), corroborating the ACSL3-mediated decrease in collagen deposition (fig. S4H).

Knockdown of *Pai-1* in KP cells did not affect cancer cell proliferation compared to the shRNA control in vitro (fig. S4, I and J). However, when we orthotopically injected the pancreata of immunocompetent mice with the KP cells transduced with either a vector control or an shRNA against *Pai-1*, we found that the suppression of *Pai-1* reduced tumor growth, as shown by the reduced pancreata weight and reduced both cancer cell and stromal fibroblast proliferation (Fig. 4L and fig. S4K). Notably, confirming the profibrotic role of PAI-1, we found a marked reduction in tumor fibrosis as evidenced by Masson trichrome staining (Fig. 4M). Moreover, *Pai-1* knockdown decreased the number of tumor alternatively activated macrophages (F4/80^+^/Arg1^+^) and T_regs_ (CD4^+^/FoxP3^+^) with a concomitant increase in CTL cells expressing granzyme B, suggesting a stronger antitumor cytotoxicity (Fig. 4, N and O, and fig. S4, L and M). Together, these data indicate that tumor-derived PAI-1 mediates fibrosis, immunosuppression, and progression of PDAC.

### *PAI-1* expression level correlates with the levels of fibrosis and immunosuppression markers and predicts poor PDAC patient survival

**Fig. 5:**
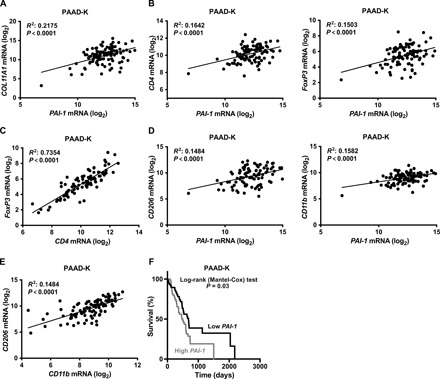
PAI-1 correlates with immunosuppression and PDAC patient survival. (**A** and **B**) Correlation between the expression level of fibrosis marker *COL11A1* (A) or T_reg_ markers *CD4*, *FoxP3* (B), and *PAI-1* expression level in subset PAAD-K from TCGA (only KRAS-mutant samples); *n* = 94. (**C**) Correlation between *FoxP3* and *CD4* expression level in human subset PAAD-K samples as in (A); *n* = 94. (**D**) Correlation between the expression level of macrophage markers *CD206* (M2 macrophages marker) or *CD11b* and *PAI-1* expression in subset PAAD-K samples as in (A); *n* = 94. (**E**) Correlation between the expression level of *CD206* and *CD11b* in human subset PAAD-K samples as in (A); *n* = 94. (**F**) Kaplan-Meier plot showing survival of human PDAC patient stratified in high (*n* = 62) and low (*n* = 62) *PAI-1* mRNA level from subset PAAD-K (only KRAS-mutant samples). Correlations were performed using the Pearson’s correlation analysis.

To confirm the relevance of PAI-1 signaling in human PDAC, we assessed the correlation between the expression level of *PAI-1* and markers of fibrosis and immunosuppression in human pancreatic tumor patients’ samples (cohort PAAD-K). Our analysis revealed a positive correlation between the expression level of *PAI-1* and *COL11A1,* a gene mediating extracellular matrix remodeling (Fig. 5A). Next, we found a positive correlation between the expression level of *PAI-1* and markers of T_regs_, *CD4*, and *FoxP3* (Fig. 5B). Moreover, our analysis evidenced a positive correlation between the expression levels of *CD4* and *FoxP3* (Fig. 5C). Thus, our results suggest an increase of immunosuppressive T_reg_ population in high *PAI-1*–expressing tumors. Further supporting the immunosuppressive role of PAI-1 in PDAC, our data evidenced a positive correlation between the expression levels of *PAI-1* and those of *CD11b* (pan-macrophage marker) or *CD206* (M2 macrophage marker), as well as between the expression levels of these two macrophage markers (Fig. 5, D and E). These results indicate a higher tumoral infiltration of M2 macrophages in tumors with higher *PAI-1* expression levels. Adding further value to our findings, our analysis evidenced that the survival rate of patients with high *PAI-1*–expressing tumors was significantly lower compared to that of patients with low *PAI-1*–expressing tumors (Fig. 5F).

### PAI-1 inhibition enhances the responsiveness of PDAC tumors to chemo- and immunotherapy

**Fig. 6:**
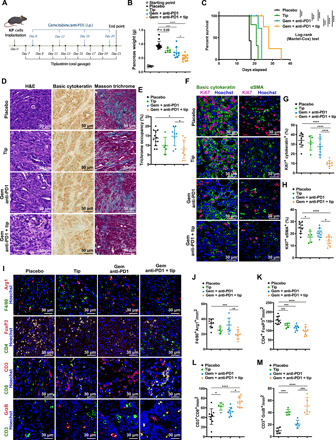
PAI-1 inhibition enhances the responsiveness of PDAC tumors to chemo- and immunotherapy. (**A**) Treatment scheme of mice bearing established orthotopic KP tumors treated as indicated. i.p., intraperitoneally. (**B**) Pancreas weight at the study end point of mice treated as in (A). Starting point: Weight of the pancreas 7 days after KP cell injection; *n* per group = 5/14/5/9/12. (**C**) Kaplan-Meier plot showing survival of mice treated as in (A); *n* per group = 7/7/5/5. (**D** and **E**) Representative images of H&E, IHC for basic cytokeratin and trichrome staining (D), and trichrome quantification reported as % of area occupancy over tumor area (E) of pancreatic tissue sections of mice treated as in (A); *n* per group = 12/5/8/9. (**F** to **H**) Representative immunofluorescence costaining (F) and quantification (G and H) of basic cytokeratin/Ki67, αSMA/Ki67, and Hoechst (nuclei) from KP orthotopic tumor sections of mice treated as in (A); *n* per group = 9/5/9/9. (**I** to **M**) Representative images of immunofluorescence costaining (I) and quantifications of F4/80^+^, arginase I^+^ (Arg1) (J), FoxP3^+^ and CD4^+^ (K), CD3^+^ and CD8^+^ T cells (L), and CD3^+^/granzyme B^+^ cells (M) from KP orthotopic PDAC mouse model sections treated as in (A); *n* per group = 9/5/9/9. In (E), (G), (H), and (J) to (M), quantifications are the average of 15 pictures per mouse. Gem, gemcitabine; Tip, tiplaxtinin. Error bars represent mean ± SD; statistical analysis was performed using one-way ANOVA. *P < 0.05, **P < 0.01, ***P < 0.001, and ****P < 0.0001.

Our results suggest that the ACSL3–PAI-1 pathway is a crucial node in PDAC progression by promoting fibrosis and immunosuppression. To give therapeutic value to our findings, we sought to combine PAI-1 inhibition with chemotherapy and checkpoint immunotherapy. To this aim, we established syngeneic orthotopic tumors derived from KP cancer cells, and we tested whether the treatment with tiplaxtinin, an orally available and selective PAI-1 inhibitor (*33*), could improve the efficacy of a combination therapy using standard chemotherapy (gemcitabine) and immunotherapy with PD-1 (programmed cell death protein 1) antagonists (anti-PD1) (Fig. 6A). Notably, in vitro treatment of cancer cells with tiplaxtinin did not affect cell proliferation (fig. S5A). We found that treatment of mice with tiplaxtinin or gemcitabine/anti-PD1 for 2 weeks led to a modest antitumor response as evidenced by the pancreas weight and overall mice survival compared to the placebo-treated mice (Fig. 6, B and C). However, the treatment with gemcitabine/anti-PD1 in combination with tiplaxtinin suppressed tumor weight and improved the overall survival of mice (Fig. 6, B and C). Notably, the number of lung metastases was significantly suppressed only in tiplaxtinin/gemcitabine/anti-PD1–treated compared to the placebo-treated mice (fig. S5, B and C). The reduced metastasis coincided with a reduction in vessel density in the tiplaxtinin/gemcitabine/anti-PD1–treated mice (fig. S5D). Staining of mouse tumor tissue with Masson trichrome evidenced reduced fibrosis in the tiplaxtinin/gemcitabine/anti-PD1–treated mice compared to placebo-, tiplaxtinin-, and gemcitabine/anti-PD1–treated mice (Fig. 6, D and E). Moreover, both tumor and stromal cells from tiplaxtinin/gemcitabine/anti-PD1–treated mice showed reduced proliferation and increased apoptosis as evidenced by Ki67 and TUNEL assay, respectively (Fig. 6, F to H, and fig. S5E).

Next, we found that the tiplaxtinin/gemcitabine/anti-PD1 treatment led to a reduction in the number of tumor M2 macrophage infiltration as shown by the reduced number of macrophages coexpressing F4/80 and Arg1 (F4/80^+^/Arg1^+^) compared to the placebo treatment (Fig. 6, I and J, and fig. S5F). Notably, the gemcitabine/anti-PD1–treated mice showed no change in the number of tumor-infiltrated macrophages, suggesting a PAI-1–specific effect (Fig. 6J). Moreover, the tumors of the tiplaxtinin-, gemcitabine/anti-PD1–, and tiplaxtinin/gemcitabine/anti-PD1–treated mice displayed reduced infiltration of T_regs_ compared to the placebo (Fig. 6, I and K) with a concomitant increase in granzyme B^+^ cytotoxic CD8^+^ immune cell population (Fig. 6, I, L, and M). Collectively, these data suggest that combination of gemcitabine with PD1 and PAI-1 inhibition may be an effective therapeutic approach to reduce fibrosis and immunosuppression and increase tumor-specific immunity in PDAC.

## DISCUSSION

The highly desmoplastic stroma and extensive tumor infiltration by immunosuppressive cell populations are considered key attributes of PDAC progression and resistance to therapy (*34*–*36*). Thus, deciphering the mechanisms by which PDAC engages its stromal fibrotic and immunosuppressive components is of great scientific interest and potential translational importance. In this study, we identified the ACSL3–PAI-1 signaling axis as a critical regulator of desmoplasia and immunosuppression in PDAC. We showed that ACSL3 contributes to PDAC progression by driving the production and secretion of the profibrotic protein PAI-1 from tumor cells. Either *Acsl3* knockout or suppression of tumor-derived *Pai-1* hinders the expansion of cancer-associated fibroblasts and extracellular matrix deposition and hampers the infiltration of immunosuppressive cell populations in tumors, including M2-like macrophages and T_regs_.

Previous evidence suggests that knockdown of *ACSL3* in primary hepatocytes decreases the activity of several lipogenic transcription factors including sterol regulatory element-binding protein-1c (SREBP1c), a positive regulator of TGF-β signaling (*37*, *38*). PAI-1 is a TGF-β–responsive gene, suggesting that ACSL3 may regulate PAI-1 through TGF-β. Future studies are warranted to understand the role of ACSL3 in TGF-β signaling pathway in pancreatic cancer.

The effects of stromal ablation in PDAC remain controversial. Recent studies provided evidence that stromal elements can act by restraining PDAC rather than supporting its progression. For instance, Hedgehog deletion specifically in tumors of a mouse model of pancreatic cancer (*Pdx1-Cre*;*Kras^LSL-G12D/+^*;*p53^fl/+^*) decreased stromal content but resulted in more aggressive, poorly differentiated, and highly vascularized tumors (*39*). Moreover, depletion of αSMA^+^ fibroblasts during pancreatic cancer development accelerated disease progression and decreased mouse survival (*40*). Furthermore, other studies highlighted the therapeutic potential of targeting other components of desmoplastic stroma such as PSCs (*11*, *41*). In this context, during activation, PSCs undergo extensive lipid metabolic remodeling and secrete lysolipids to support PDAC progression (*11*). Given the avidity of mutant KRAS for extracellularly derived fatty acids (*10*, *42*, *43*), it would be of interest to assess whether ACSL3 is indispensable for the activation of lipids released by activated PSCs in PDAC. Related to this, PAI-1 has been shown to contribute to the activation of PSCs (*44*), hinting toward a feedback loop that sustains PSC activation. Accordingly, our work reveals that it is specifically the tumor-derived PAI-1 that promotes the tumor immunosuppressive microenvironment and fibrosis leading to increased PDAC progression.

In solid tumors such as non–small cell lung cancer and kidney cancer, immunotherapy using immune checkpoint–blocking antibodies has shown remarkable clinical efficacy (*45*, *46*). In contrast, immunotherapy has been proven to be ineffective for pancreatic cancer treatment and gemcitabine remains the main treatment option (*47*, *48*). The reasons for the lack of efficacy are still unclear but could be caused by the excessive desmoplasia and poor immunogenicity (*49*, *50*). Here, we assessed the efficacy of a combination therapy using standard chemotherapy (gemcitabine) and immunotherapy (anti-PD1) with tiplaxtinin, an orally available inhibitor of PAI-1 in a mouse model of PDAC. Our data show that the treatment with tiplaxtinin caused a marked reduction of tumor fibrosis and immunosuppression, while it enhanced cytotoxic T cell response. This effect was associated with increased efficacy of chemotherapy/immunotherapy as shown by stronger suppression of tumor growth and metastases formation as well as increased overall mice survival. Thus, our data reveal PAI-1 as a druggable target for PDAC treatment.

We found increased levels of activated CD8^+^ tumor-infiltrating T cells despite the fact that we detected reduced CD31^+^ blood vessel density. However, we revealed a strong reduction of immunosuppressive cell populations that can explain the increased CD8^+^ T cell tumor infiltration. In contrast, a recent study revealed that combination of mitogen-activated protein kinase kinase (MEK) and cyclin-dependent kinase 4/6 (CDK4/6) inhibitors triggers senescence-associated secretory phenotype (SASP)–mediated increase in CD31^+^ cells and endothelial activation, promoting CD8^+^ T cell tumor infiltration. Notably, the infiltration of CD8^+^ T cells in PDAC occurred without any changes in immunosuppressive cell populations but was dependent on vascular cell adhesion molecule 1 (VCAM1)–dependent endothelial activation (*51*). These results suggest that qualitative changes in endothelial cell activation may have a greater impact on the immune landscape of PDAC than changes in blood vessel density.

PAI-1 inhibitors have so far provided antithrombotic benefits without causing bleeding in many preclinical models including nonhuman primates (*52*, *53*). Unfortunately, treatment with tiplaxtinin has been unsuccessful in human clinical trials for thrombosis due to an unfavorable risk to benefit ratio and the need for tight dose control to avoid provoking bleeding disorders. However, the use of tiplaxtinin, or other PAI-1 inhibitors, for cancer treatment still has to be evaluated. Our preclinical data suggest that the addition of PAI-1 inhibitors in chemotherapy and checkpoint immunotherapy holds promise for the treatment of pancreatic cancer. Fibrosis and immunosuppression are common features of many types of malignancies. Furthermore, data suggest that high levels of PAI-1 are associated with poor clinical outcomes and chemo- or targeted therapy resistance in different types of cancer (*54*–*57*). Therefore, further trials are warranted to validate similar combination strategies with PAI-1 inhibitors for the treatment of other cancer types.

## MATERIALS AND METHODS

### Experimental models

#### *Cell lines*

HPDE-i*KRAS*^G12D^ cell line was a gift of K. L. Scott (*26*). The HPDE-i*KRAS*^G12D^ cell line was DNA fingerprinted for provenance and screened for mycoplasma and cultured in an incubator at 37°C and 5% CO_2_ for one passage in KSFM (keratinocyte serum-free media) (Gibco) and supplied with epidermal growth factor and bovine pituitary extract as prescribed by Gibco. Subsequently, cells were transferred in RPMI 1640 (Gibco) supplied with 10% fetal bovine serum (FBS) and penicillin/streptomycin for at least two passages. Expression of *KRAS^G12D^* was induced by administration of Dox (Fisher Scientific, BP2653) in the culture medium to a concentration of 500 ng/ml for 24 hours.

The KPC/WCB3 cell line (C57BL/6J background) used for the KP orthotopic PDAC mouse model was obtained from Ximbio on behalf of Cancer Research Technology Ltd. and cultured in Dulbecco’s Modified Eagle medium (DMEM) (Gibco) supplied with 10% FBS and penicillin/streptomycin in an incubator at 37°C and 5% CO_2_.

#### *Human studies*

The patient-derived frozen PDAC samples were provided by the Institute of Pathology, Translational Research Unit. All the patients were females. The TMA was provided by E. Karamitopoulou, Institute of Pathology, University of Bern. The use of human samples was approved by the ethics commission (Swissethics), ID: 2017-01322. All samples were provided upon patient consent.

#### *Mice*

The mixed background tamoxifen-inducible *LSL-Kras^G12D/+^*;*Trp53^fl/fl^*;*Pdx1Cre^ERT2^*;*Acsl3^−/−^* mouse model was obtained by crossing the strain Tg(Pdx1-cre/Esr1*)#Dam/J (*15*) (from The Jackson Laboratory, stock number 024968) with *B6.129SS4-kras^tm4Tyj^/J* (*13*) (from The Jackson Laboratory, stock number 008179), *B6.129P2-Trp53^tm1Brn^/J* (*14*) (from The Jackson Laboratory, stock number 008462), and *B6;129S5-Acsl3Gt(OST148301)Lex/Orl* (constitutive *Acsl3^−/−^* mouse, obtained from the European Mouse Mutant Archive). The *B6;129S5-Acsl3Gt(OST148301)Lex/Orl* mice carry a gene trap disrupting *Acsl3* upstream of exon 1. Mice were backcrossed for 12 generations, before creating the experimental groups. The constitutive prenatal pancreas *Cre*-expressing *LSL-Kras^G12D/+^*;*Trp53^flfl^*;*Pdx1-Cre^6Tuv^*;*Acsl3^−/−^* mouse model was obtained by crossing strain *B6.FVB-Tg(Pdx1-cre)6Tuv/J* (*18*) (from The Jackson Laboratory, stock number 014647) with *B6.129SS4-kras^tm4Tyj^/J* (from The Jackson Laboratory, stock number 008179), *B6.129P2-Trp53^tm1Brn^/J* (from The Jackson Laboratory, stock number 008462), and *B6;129S5-Acsl3Gt(OST148301)Lex/Orl*. Mice were backcrossed for eight generations before creating the experimental groups. For the KP orthotopic PDAC mouse model, we used the strain *C57BL/6J* (Charles River Laboratories). Only male littermates were used for the experiments. Animal handling and experimental procedures were performed in compliance with the federal guidelines and were approved by the Veterinaerdienst des Kantons Bern. The full list of all mouse strains and cell lines used can be found in table S1.

### Reagents and plasmids

Induction of the expression of *KRAS^G12D^* was performed by supplementing cell culture medium with Dox (500 ng/ml; Fisher Scientific, BP2653). For lentiviral production, the pCMV-VSV-G (Addgene, plasmid #8454) and pCMV-dR8.2 dvpr (Addgene, plasmid #8455) and pLKO.1 hygro (Addgene plasmid #24150) plasmids were a gift from B. Weinberg (*58*). The human ACSL3-containing plasmid (pEGFP-C1 ACSL3_HA_) used to reexpress ACSL3 upon knockdown was provided by J. Füllekrug (*59*).

### shRNAs, virus production, and transduction

The validated shRNAs were obtained as bacterial glycerol stock from Sigma-Aldrich. Recombinant lentiviruses were produced by transfecting human embryonic kidney (HEK) 293T cells, using the TransIT-293 Transfection Reagent (Mirus, MIR2705), with pCMV-VSV-G (VSV-G protein), pCMV-dR8.2 (lentivirus packaging vector), and lentiviral constructs, according to the manufacturer’s instructions. The full list of the shRNA sequences used in this manuscript can be found in table S1.

### Animal studies

Mice were maintained under controlled humidity and temperature conditions, with a standard 12-hour light/12-hour dark cycle, and were fed ad libitum. Genomic DNA extraction and PCR assay were performed using the KAPA HotStart Mouse Genotyping Kit (Kapa Biosystems, KK7352) and KAPA2G Fast HotStart Genotyping Mix (Kapa Biosystems, KK5621), respectively, according to the manufacturer’s instructions. The mice genotypes were confirmed following the corresponding The Jackson Laboratory protocols. The PCR for the *Acsl3* genotyping was performed according to the KAPA HotStart Mouse Genotyping Kit with an annealing temperature at 60°C. The full list of oligos used to genotype the mice can be found in table S1. Recombination in *KPC* mice was induced with five consecutive daily administrations of tamoxifen with intraperitoneal injection at 3 weeks of age. The injected solution contained tamoxifen presolubilized in ethanol and diluted in sunflower oil (Sigma-Aldrich) with a proportion of 1:9 parts ethanol/sunflower oil. Tamoxifen (2.5 mg) was administered per mouse with each injection. The *KPC* mice were sacrificed 8 weeks after induction (11 weeks of age), while the *KPCC* mice were sacrificed at 6 weeks of age. Pancreata were retrieved after anesthesia and perfusion of the animal with 20 ml of phosphate-buffered saline (PBS).

For the orthotopic KP model, we injected 200,000 cells per mouse directly in the pancreas as previously described (*60*). Tiplaxtinin (Tocris Bioscience) was presolubilized in dimethyl sulfoxide (DMSO) (stock concentration, 40 mg/ml) administered at a dose of 20 mg/kg with oral gavage every second day. After presolubilization in DMSO (Sigma-Aldrich), the vehicle used for dilution was corn oil (Sigma-Aldrich) to a total volume of 100 μl per mouse. Gemcitabine was administered at a dose of 60 mg/kg with intraperitoneal injection every fourth day diluted from a stock (50 mg/ml) in PBS (total volume injected, 100 μl per mouse). The anti-PD1 monoclonal antibody (mAb) and isotype control (Bio X Cell) were diluted in PBS and administered at a dose of 200 μg per mouse via intraperitoneal injection every fourth day (100 μl per mouse). Tiplaxtinin was administered every other day, while gemcitabine and αPD1 mAb were administered every 4 days. The treatment scheme included a total of eight administrations of tiplaxtinin and four administrations of gemcitabine and anti-PD1 mAb. Mice were sacrificed 21 days after KP cell implantation. In the *Pai-1* knockdown KP orthotopic model, the mice were sacrificed 15 days after KP cell implantation.

The lung metastases were quantified from the average of three tissue sections of paraffin-embedded and H&E-stained whole lung tissue separated by approximately 30 μm from each other. The number of metastases was expressed as the number of metastases per lung.

### Reverse transcription PCR

RNA was extracted using the RNeasy Kit (Qiagen, 74104), and complementary DNA (cDNA) was synthesized with the RevertAid First-Strand cDNA Synthesis Kit (Thermo Fisher Scientific, K1622). qPCR was performed in 96-well plates (TreffLab) with Fast SYBR Green (Thermo Fisher Scientific, 4367659). The normalization was performed with the ΔΔ*C*_T_ method. The full list of the oligonucleotides used can be found in table S1.

### Histology

Tissues for histology were fixed at 4°C overnight in 4% paraformaldehyde in PBS before paraffin embedding. All sections used for histological analysis were 5 μm thick. Histological characterization and consequent scoring of neoplastic lesion grade of H&E-stained sections of pancreata were done with supervision and confirmation from a pathologist. Tumor burden was assessed by digital quantification of the area occupied by tumors compared to unaffected tissue using QuPath v.0.1.2.

### Immunohistochemistry

IHC was conducted on paraffin-embedded tissue. Sections were deparaffinized, rehydrated through a graded series of ethanol solutions, and subjected to antigen retrieval by boiling for 12 min in antigen retrieval sodium citrate buffer (pH 6). Sections were then pretreated for 30 min with 3% hydrogen peroxide (Sigma-Aldrich, 216763) in PBS, washed twice with 0.1 M tris-buffered saline (TBS), blocked for 1 hour in 2% bovine serum albumin (BSA) in TBS containing 0.1% polysorbate 20 (TBS-T) and 10 min in 2.5% normal horse serum (Vector Laboratories, #S-2012), and incubated with primary antibodies diluted appropriately in blocking solution. The following day, sections were washed in TBS-T and incubated with ready-to-use secondary antibody (Vector Laboratories, #MP-7401) for 1 hour or with Mouse on Mouse (M.O.M.) ImmPRESS Peroxidase Polymer Anti-Mouse Reagent (Vector Laboratories, #MPX-2402) for 10 min, and the staining was revealed with DAB+ solution (Dako, #Κ3467). Tissue sections were then counterstained with methyl green (Vector Laboratories, #H-3402), dehydrated, and mounted. Stained sections were scanned using a Panoramic MIDI II digital slide scanner (3DHISTECH Ltd). Image analysis was performed using QuPath (open source software, v.0.1.2).

### TMA staining and scoring

The TMA IHC staining with anti-ACSL3 (Thermo Fisher Scientific, catalog no. PA5-29507) was performed as previously described (*10*). The antigen retrieval method consisted of a 20-min, 95°C incubation in a sodium citrate buffer solution at pH 6. The antibody dilution was 1:300, and the incubation time was 30 min at room temperature. The TMA scoring was done by a pathologist with experience on human pancreatic cancer (E.K.). We scored for low, intermediate, or high ACSL3 protein levels based on a histological score that takes into consideration the percentage of cells stained at different intensities (H-score). We considered an H-score of less than 100, from 100 to 200, and above 201 having a low, intermediate, or high staining intensity, respectively. The scoring of fibrosis was done by quantification of Masson trichrome–positive tissue with the QuPath software. The blue RGB channel area was automatically analyzed and quantified with the quantification module and expressed as mean pixel intensity (occupancy weighted by staining intensity).

### Immunofluorescence

For immunofluorescence conducted on paraffin-embedded tissue, all stainings with primary antibodies were done after deparaffinization, rehydration through a graded series of ethanol solutions, antigen retrieval by boiling for 10 min in sodium citrate buffer (pH 6), and blocking in 2% BSA in PBS-T buffer. Antibodies were diluted in blocking solution, and antibody incubation was done at 4°C overnight. Secondary fluorescent-tagged antibodies were from molecular probes (Invitrogen). Stained cells and sections were scanned using a Panoramic MIDI II digital slide scanner (3DHISTECH Ltd.) or with a confocal microscope (Carl Zeiss). Image analysis was performed with Imaris software (www.bitplane.com).

### Immunoblotting

Cells were lysed in radioimmunoprecipitation assay (RIPA) buffer [50 mM tris-HCl (pH 8.0), 150 mM NaCl, 1.0% NP-40, 0.5% sodium deoxycholate, and 0.1% SDS] containing complete EDTA-free protease inhibitors (Roche) and 1 mM phenylmethylsulfonyl fluoride. Human samples were first pulverized in a liquid nitrogen mortar and homogenized in RIPA buffer before lysis incubation. Samples were resolved by SDS–polyacrylamide gel electrophoresis in Bio-Rad blotting chamber, transferred to nitrocellulose membrane using a semidry chamber (Bio-Rad), and blocked in 5% BSA. Membranes were then incubated overnight at 4°C with primary antibody diluted in 5% BSA in PBS containing 0.1% Tween. Secondary fluorescent-tagged antibodies were from LI-COR Biosciences, and development was done in LI-COR fluorescence-chemiluminescence detector. All antibodies and their dilutions are listed in table S1.

### FACS analysis

For the flow cytometry experiment, 40 μl of blood was taken with a capillary glass tube from the tail of each mouse. Blood was kept on ice and immediately centrifuged at 1400 rpm for 7 min. The supernatant was removed, and the residual cells were resuspended in 100 μl of fluorescence-activated cell sorting (FACS) buffer (PBS supplied with 2% FBS). Red blood cells were osmotically lysed with the RBC lysis buffer (BioLegend, #420301) according to the manufacturer’s instructions. Cells were then centrifuged at 1400 rpm for 7 min, and the supernatant was removed and washed three times with 1 ml of FACS buffer. Subsequently, they were resuspended in 100 μl of blocking buffer for 30 min on ice. The blocking buffer consisted of one-third supernatant from HB-197 cells [Fc (fragment crystallizable) region blocker], one-third FACS buffer, and one-third of equally mixed rat, hamster, rabbit, mouse, and FBS. Antibodies were added directly to the sample and incubated for 1 hour on ice in the dark. Afterward, cells were centrifuged once at 1400 rpm for 7 min, the supernatant was removed, and the cell pellet was washed three times with 1 ml of FACS buffer. The cell pellet was resuspended in 100 μl of FACS buffer and immediately measured with a BD FACSVerse. Cells were gated according to their forward and side scatter values to exclude cell clusters. CD45^+^ events were recorded and subgated as shown in fig. S3B.

### ACSL enzymatic activity assay

The ACSL activity was measured in homogenate and cell fractions by a radioisotopic assay of labeled palmitate incorporation into palmitoyl CoA. The assay mixture contained 175 mM tris-HCl (pH 7.4), 8 mM MgCl_2_, 5 mM dithiothreitol, 1 mM adenosine triphosphate (ATP), 0.2 mM CoASH, and 50 μM palmitate containing 0.3 Ci of [^3^H]palmitic acid in a solution of 0.5 mM Triton X-100 with 10 μM EDTA. The reaction was initiated by addition of 10 to 30 μg of cell fraction protein. The total volume was 200 μl in each assay. The reaction was terminated after 10 min at room temperature by addition of 1 ml of Dole’s reagent (isopropanol:heptane:1 M H_2_SO_4_, 40:10:1 by volume). Heptane (2 ml) and H_2_O (0.5 ml) were added, and the upper layer was removed. The lower layer was washed with 2 ml of heptane, and the top phase was removed. Scintillation mixture contained scintillation fluid and lower phase in a 1:1 ratio, and it was read in the volume of 100 μl on a white 96-well plate (PerkinElmer) with a scintillation plate reader (Packard Bioscience). ACSL activity is expressed as nanomoles per milligram of protein per minute.

### Cytokine array

The Cayman Human cytokine array (catalog no. ARY005) and the Mouse cytokine array (catalog no. ARY028) were used to perform the assays. In both cases, 150,000 cells were plated in a six-well plate, and the day after, cell culture medium was refreshed. Supernatant from cultured cells was collected after 24 hours of incubation, and the assay was performed according to the manufacturer’s instructions. Acquisition of the array image was performed with the LI-COR instrument on the chemiluminescent channel, and optical density was quantified with the Image Studio software (www.licor.com).

### Colocalization analysis

The analyses were performed using the Imaris Studio Software. To exclude false positivity because of the background of the green channel, a first mask was set on it, with a threshold value of “1” for bright intensity. The analysis was run with the Imaris CoLoc and Imaris Cell identification tools, and the results are the average of at least 15 pictures per mouse section. The number of mice per group is indicated in the figure legends. For the colocalization between basic cytokeratin and Ki67, we run the algorithm “cell identification”; for the value “nucleus,” the blue channel [4′,6-diamidino-2-phenylindole (DAPI)] was selected; for the value “positive,” the red channel (Ki67) was selected; and for the “membrane” value, the green channel (basic cytokeratin) was selected.

For the colocalization between αSMA and Ki67, we run the algorithm cell identification, and we selected the blue channel (DAPI) for the nuclei; for the value positive, the red channel (Ki67) was selected; and for the membrane value, the green channel (αSMA) was selected. The results of at least 15 pictures were averaged for every mouse and plotted. Tumor microvessel density was quantified on CD31-stained sections by following the Weidner method, and CD31 occupancy was quantified with the open source software QuPath.

### Database analysis

Data from the TCGA dataset PAAD were downloaded from the web GUI Xena Browser. They are expressed as RSEM (RNAseq by expectation-maximization) normalized count (native format of the dataset). For the correlations and survival analysis of the dataset PAAD, only *KRAS*-mutated tissue samples were considered (subset PAAD-K). Patients with partial or incomplete data were eliminated from the analysis. The patient number is reported in the related legends. For the division in high and low expression, the median was considered as threshold to discriminate between the two groups. The data used in the comparative analysis of *ACSL3* expression in normal tissue, primary PDAC, and metastatic tissue were downloaded from the GEO, subset GSE71729, and, in the paired analysis of *ACSL3* expression comparison between PDAC and healthy matched tissue, from the subset GSE62452. In both cases, data were expressed as entire numbers: in the first case normalized to one, and in the second by global average expression.

### Statistical analysis and elaboration

Error bars represent mean ± SD or mean ± SEM (indicated in the figure legends). The significance of the results was determined by using two-tailed unpaired Student’s *t* test or one-way analysis of variance (ANOVA) when more than two groups were compared. Significance is indicated in the related figure legends. The in vitro experiments (except the two cytokine arrays that were repeated twice) were repeated three times. No outliers were found in any dataset, and no animals or data were excluded from statistical analysis. Correlations are expressed as *R* (Pearson), and fitting is done as implemented in GraphPad Prism v.7.00 (GraphPad Software, San Diego, CA).

## Supplementary Material

abb9200_Table_S1.xlsx

abb9200_SM.pdf

## SUPPLEMENTARY MATERIALS

Supplementary material for this article is available at http://advances.sciencemag.org/cgi/content/full/6/44/eabb9200/DC1

## Appendix

View/request a protocol for this paper from *Bio-protocol*.
