# Supplementary material for: ACSL3–PAI-1 signaling axis mediates tumor-stroma cross-talk promoting pancreatic cancer progression
Source: Sci Adv. 2020 Oct 30;6(44):eabb9200. doi: 10.1126/sciadv.abb9200 (PMC7608806; doi:10.1126/sciadv.abb9200)
Supplement: abb9200_SM.pdf [file abb9200_SM.pdf]

Science Advances
AAAS

advances.sciencemag.org/cgi/content/full/6/44/eabb9200/DC1

# Supplementary Materials for

## ACSL3–PAI-1 signaling axis mediates tumor-stroma cross-talk promoting pancreatic cancer progression

Matteo Rossi Sebastiano, Chiara Pozzato, Maria Saliakoura, Zhang Yang, Ren-Wang Peng, Mirco Galiè, Kevin Oberson, Hans-Uwe Simon, Evanthia Karamitopoulou, Georgia Konstantinidou*

*Corresponding author. Email: georgia.konstantinidou@pki.unibe.ch

Published 30 October 2020, *Sci. Adv.* **6**, eabb9200 (2020)
DOI: 10.1126/sciadv.abb9200

**The PDF file includes:**

Figs. S1 to S5

**Other Supplementary Material for this manuscript includes the following:**

(available at advances.sciencemag.org/cgi/content/full/6/44/eabb9200/DC1)

Table S1

# SUPPLEMENTARY FIGURES AND FIGURE LEGENDS

Supplementary Figure 1 Rossi Sebastiano M. et al.

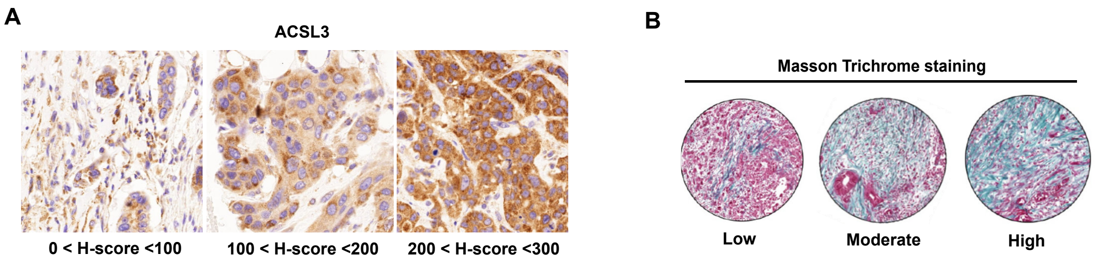


**Supplementary Figure 1. ACSL3 is overexpressed in pancreatic ductal adenocarcinoma.**

**(A)** Representative IHC staining of ACSL3 from a human tissue microarray showing low H-score (0<H-score<100), intermediate (100<H-score<200) and high (200<H-score<300).

**(B)** Representative Masson trichrome staining of pancreatic cancer tissue from a human tissue microarray showing low, intermediate or high staining.

**Supplementary Figure 2 Rossi Sebastiano M. et al.**

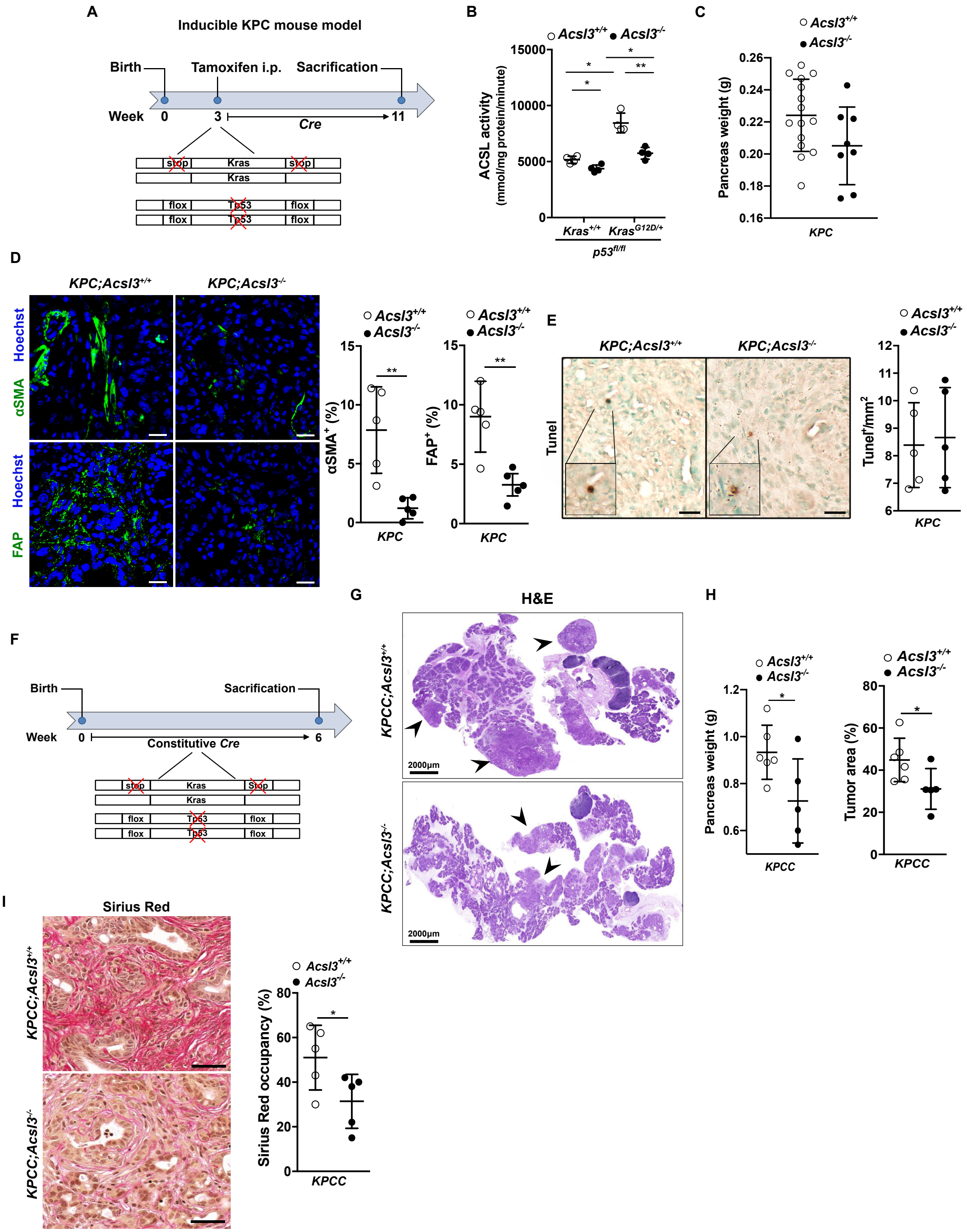

**Supplementary Figure 2. *Acsl3* knockout reduces tumor fibrosis and suppresses tumor progression.**

**(A)** Scheme representing the protocol followed for the induction of *LSL-Kras*$^{G12D/+}$*;p53*$^{flox/flox}$*;Pdx1-Cre*$^{ERT2}$ (KPC) mice.

**(B)** ACSL activity from mouse pancreata of the following genotypes (left to right): *p53*$^{flox/flox}$*;Pdx1-Cre*$^{ERT2}$*;Acsl3*$^{+/+}$, *p53*$^{flox/flox}$*;Pdx1-Cre*$^{ERT2}$*;Acsl3*$^{-/-}$, *LSL-Kras*$^{G12D/+}$*;p53*$^{flox/flox}$*;Pdx1-Cre*$^{ERT2}$*;Acsl3*$^{+/+}$, *Kras*$^{G12D/+}$*;p53*$^{flox/flox}$*;Pdx1-Cre*$^{ERT2}$*;Acsl3*$^{-/-}$ mouse pancreas samples (n=4 mice/per group).

**(C)** Pancreas weight of *KPC;Acsl3*$^{+/+}$ (n=15) and *KPC;Acsl3*$^{-/-}$ (n=8) mice at 8 weeks after tumor onset treated as in (A).

**(D)** Representative immunofluorescence images (left) and relative quantification (right) of αSMA- and FAP-positive cells from *KPC;Acsl3*$^{+/+}$ and *KPC;Acsl3*$^{-/-}$ mouse pancreatic tumor sections. Quantifications are expressed as average of 15 pictures/mouse; n=5 mice/group. Scale bar: 50 µm.

**(E)** Representative Tunel staining images (left) and quantification of positive nuclei (right) of *KPC;Acsl3*$^{+/+}$ and *KPC;Acsl3*$^{-/-}$ mouse sections; n = 5 mice/group. Scale bar: 50 µm.

**(F)** Scheme representing the experimental protocol followed to obtain the *LSL-Kras*$^{G12D}$*;p53*$^{flox/flox}$;*Pdx1-Cre*$^{6tuv}$*;Acsl3*$^{+/+}$ (*KPCC*) mice.

**(G)** Representative hematoxylin and eosin (H&E)-stained mouse pancreas of *LSL-Kras*$^{G12D}$*;p53*$^{flox/flox}$;*Pdx1-Cre*$^{6tuv}$*;Acsl3*$^{+/+}$ (*KPCC;Acsl3*$^{+/+}$) and *LSL-Kras*$^{G12D}$*;p53*$^{flox/flox}$;*Pdx1-Cre*$^{6tuv}$*;Acsl3*$^{-/-}$ (*KPCC;Acsl3*$^{-/-}$) at 6 weeks after birth.

**(H)** Pancreas weight (left) and tumor burden quantification (% of tumor area/total pancreas area) from H&E stained mouse pancreas sections of *KPCC;Acsl3*$^{+/+}$ (n = 6 mice) and *KPCC;Acsl3*$^{-/-}$ (n = 5 mice) at 6 weeks after birth.

**(I)** Representative images of Sirius Red staining (left) and related quantification (right) of *KPCC;Acsl3*$^{+/+}$ and *KPCC;Acsl3*$^{-/-}$ mice at 6 weeks of age; Graph reports the % of area occupancy over tumor area assessed on a representative pancreas section per mouse; n = 5 mice/group. Scale bar: 50 μm.

Error bars represent mean ± SD, statistical analysis was performed using unpaired Student's t test or one-way ANOVA. * $p<0.05$, ** $p<0.01$.

Supplementary Figure 3 Rossi Sebastiano M. et al.

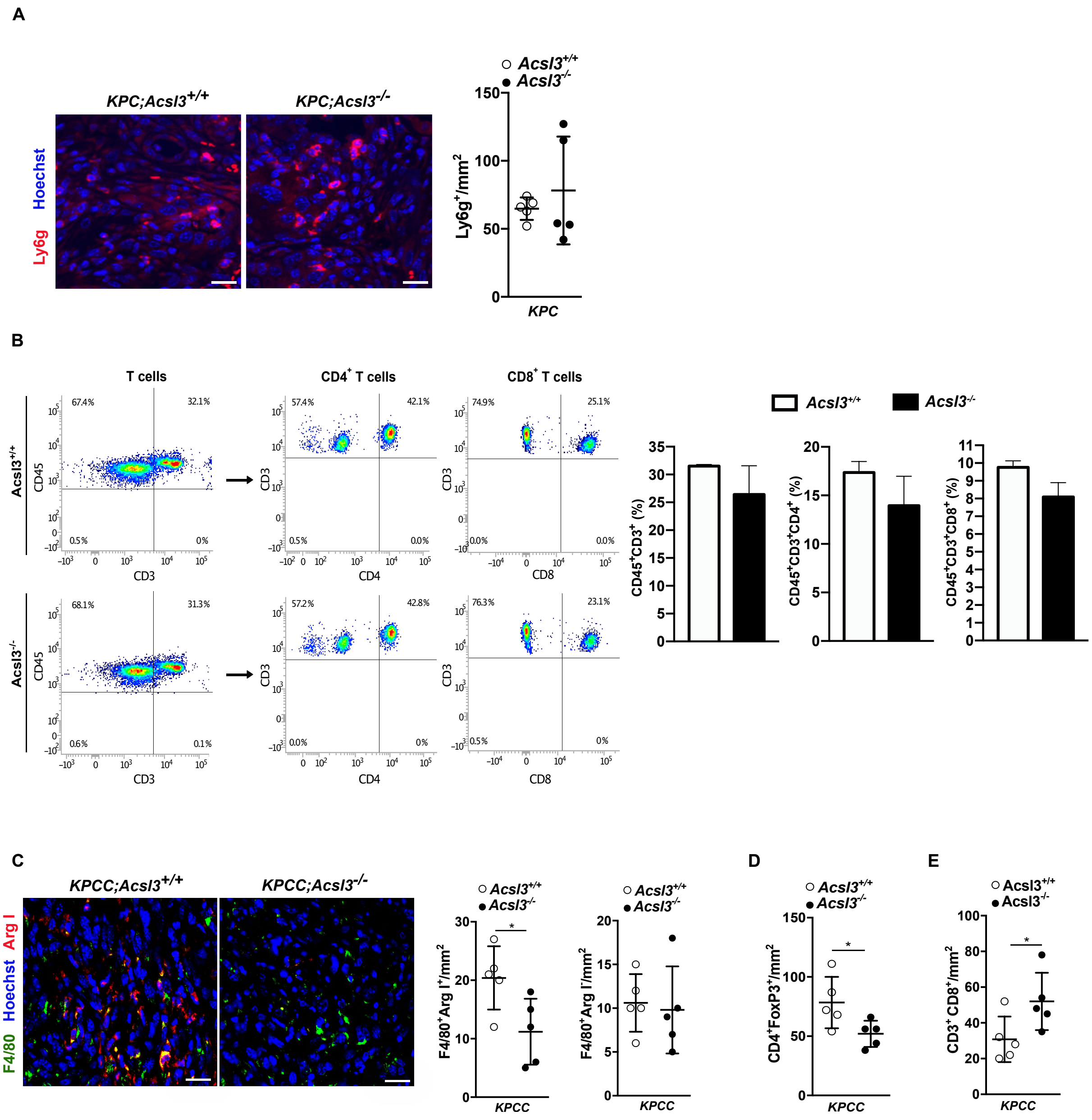


**Supplementary Figure 3. ACSL3 drives tumor microenvironment towards immunosuppression.**

**(A)** Representative immunofluorescence images of Ly6g staining (left) and relative quantification (right) from *KPC;Acsl3*$^{+/+}$ and *KPC;Acsl3*$^{-/-}$ mouse tumor sections; n=5. Scale bars: 30 µm.

**(B)** Representative density plots and gating strategy of flow cytometry staining of $CD45^{+}$, $CD3^{+}$, $CD4^{+}$ and $CD8^{+}$, markers of T cells (left) and related quantification of $CD45^{+}$, $CD3^{+}$, $CD4^{+}$ and $CD8^{+}$ T cells (right) from the blood of healthy *Pdx1-Cre*$^{ERT2}$*;p53*$^{flox/flox}$*;Acsl3*$^{+/+}$ and *Pdx1-Cre*$^{ERT2}$*; p53*$^{flox/flox}$*;Acsl3*$^{-/-}$ mice.

**(C)** Representative immunofluorescence images of F4/80 and Arg1 staining (left) and their quantification (right) from *KPCC;Acsl3*$^{+/+}$ and *KPCC;Acsl3*$^{-/-}$ mice. Quantifications are expressed as average of 15 pictures/mouse (n=5 mice/group). Scale bar: 50 µm.

**(D, E)** Quantification of $CD4^{+}$/$FoxP3^{+}$ (D), and $CD3^{+}$/$CD8^{+}$ (E) cells per $mm^2$ upon immunofluorescence staining on tumor bearing tissue of *KPCC;Acsl3*$^{+/+}$ and *KPCC;Acsl3*$^{-/-}$ mice; n=5 mice/group.

Error bars represent mean ± SD, statistical analysis was performed using unpaired Student's t test. * $p<0.05$.

Supplementary Figure 4 Rossi Sebastiano M. et al.

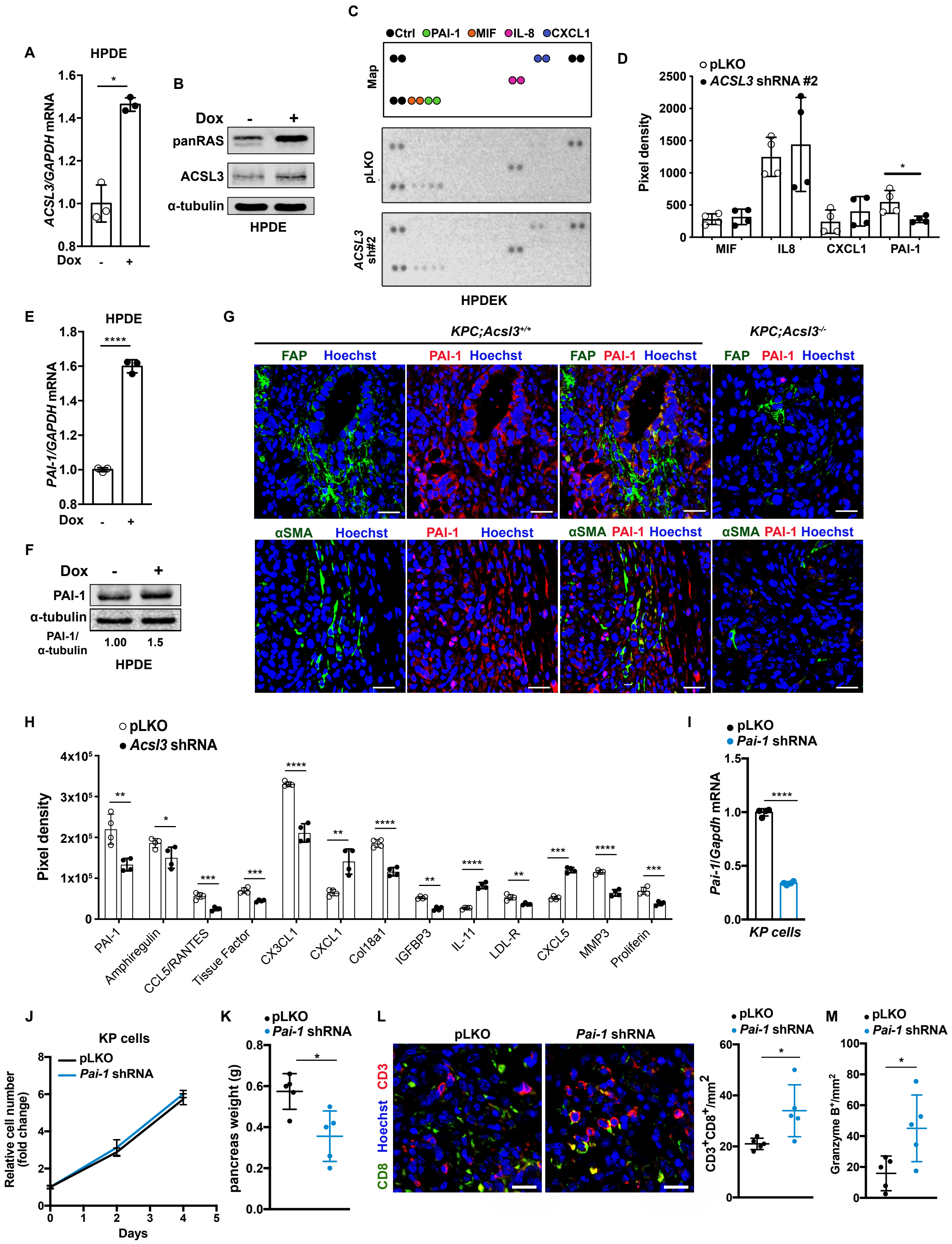

**Supplementary Figure 4. ACSL3 controls PAI-1 levels in PDAC.**

**(A)** Quantitative real time PCR for *ACSL3* in HPDE cells treated with/without doxycycline in cell culture media for 24 h (to induce $KRAS^{G12D}$ expression: HPDEK); n=3/group.

**(B)** Immunoblot for ACSL3 and total RAS on HPDE cells treated as in (A).

**(C)** Map and membrane development from cytokine array related to main Fig. 4. HPDEK cells were infected either with pLKO empty vector or with a shRNA against ACSL3 (sh#2), selected with hygromycin and plated for experiment. 24h later the supernatant was used to run the cytokine assay according to manufacturer's recommendations. This experiment was repeated twice.

**(D)** Pixel density quantification of positive hits from (C); n = 4.

**(E)** Quantitative real time PCR for *PAI-1* in HPDE cells treated as in (A).

**(F)** Immunoblot for PAI-1 in HPDE cells treated as in (B).

**(G)** Representative immunofluorescence images of FAP (green, top), αSMA (green, bottom) and PAI-1 (red) staining on tumor lesions from *KPC;Acsl3*$^{+/+}$ (n=5 mice) and *KPC;Acsl3*$^{-/-}$ mice (n=5 mice). Scale bars: 30 μm. Colocalization PAI-1/FAP: 7.61% ± 0.87 and PAI-1/αSMA: 5.23% ± 1.62 in *KPC;Acsl3*$^{+/+}$ tumors.

**(H)** Pixel density quantification of the significantly-altered mouse cytokines obtained with a mouse cytokine array. KP cells ($Kras^{G12D/+}$;$p53^{R172H}$) were transduced either with pLKO empty vector or with a shRNA against mouse *Acsl3*, selected with hygromycin and plated for experiment. 24h later the supernatant was used to run the cytokine assay according to manufacturer's recommendations; n = 4. This experiment was repeated twice.

**(I, J)** Quantitative real time PCR for *Pai-1* (J) and cell proliferation assay (K) in KP cells ($Kras^{G12D/+}$;$p53^{R172H}$) previously transduced either with a pLKO control or a shRNA against *Pai-1*; n=3. This experiment was repeated 3 times.

**(K)** Pancreas weight of mice bearing established orthotopic KP tumors 15 days after KP cell implantation. KP cells were transduced with a pLKO control or a shRNA against *Pai-1* before orthotopic implantation in mouse pancreas of syngeneic C57BL/6J mice; n=5 mice/group.

**(L)** Representative immunofluorescence images (left) and quantification (right) of $CD3^{+}$ and $CD8^{+}$ T-cell co-staining of pancreatic tumor sections from mice bearing established orthotopic KP tumors generated and treated as in (K); n=5 mice/group. Scale bars: 50 μm.

**(M)** Quantification of Granzyme $B^{+}$ cells from immunohistochemistry stained pancreatic tumor sections from KP orthotopic PDAC mouse model generated as in (K); n=5 mice/group.

Error bars represent mean ± SD, statistical analysis was performed using unpaired Student's t test. * *p<0.05, ** p<0.01, *** p<0.001, **** p<0.0001.*

**Supplementary Figure 5 Rossi Sebastiano M. et al.**

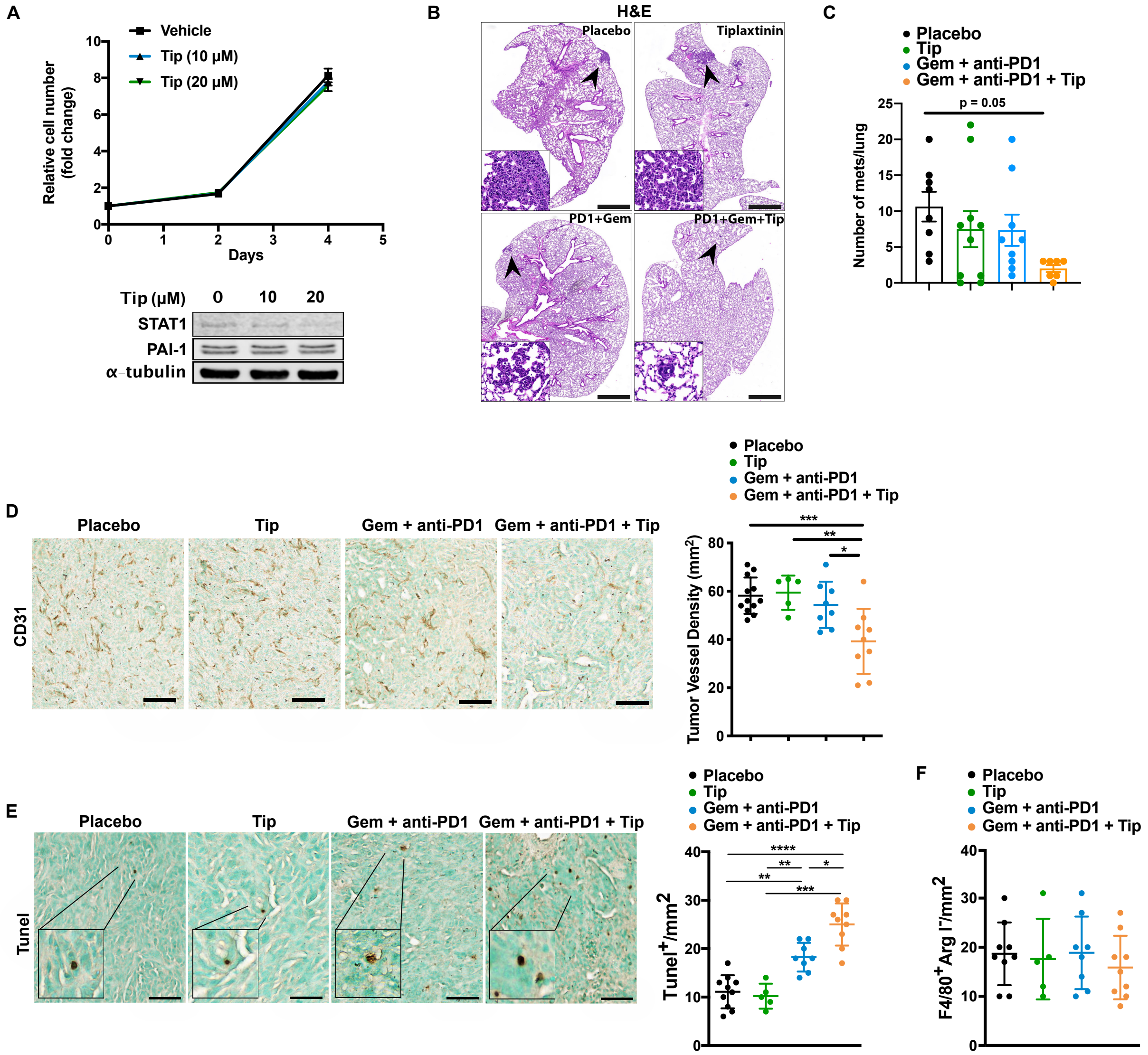


**Supplementary Figure 5. PAI-1 inhibition renders pancreatic cancer responsive to chemotherapy and checkpoint immunotherapy *in vivo*.**

**(A)** Representative cell proliferation assay (top) and immunoblot for STAT1 (bottom) showing that tiplaxtinin has no effect on cell proliferation in KP mouse cells *in vitro*, while suppresses its downstream target STAT1.

**(B)** Representative images of H&E stained lung tissue sections showing metastases to the lung of KP orthotopic PDAC mouse model treated as in (A). The arrows indicate the magnified areas. Scale bar: 1000 μm.

**(C)** Number of lung metastases of mice bearing established orthotopic KP tumors treated with placebo (n=8), tiplaxtinin (n=10), anti-PD1 mAb and gemcitabine (n=9) or anti-PD1 mAb, gemcitabine and tiplaxtinin (n=7). Gem: gemcitabine. Tip: tiplaxtinin.

**(D)** Representative immunohistochemistry staining images (left) and quantification (right) of $CD31^{+}$ vessel density from established orthotopic KP tumors treated with placebo (n=12), tiplaxtinin (n=5), anti-PD1 mAb and gemcitabine (n=8) or anti-PD1 mAb, gemcitabine and tiplaxtinin (n=9). Gem: gemcitabine. Tip: tiplaxtinin. Quantifications are the average of 15 pictures/mouse. Scale bar: 50 μm. Gem: gemcitabine; Tip: tiplaxtinin.

**(E)** Representative images (left) and quantification (right) of Tunel (apoptosis assay) staining on pancreatic tissue sections of KP orthotopic PDAC mouse model treated with placebo (n=10), tiplaxtinin (n=5), anti-PD1 mAb and gemcitabine (n=8) or anti-PD1 mAb, gemcitabine and tiplaxtinin (n=9). Gem: gemcitabine. Tip: tiplaxtinin. Scale bar: 50 μm.

**(F)** Representative quantifications based on immunofluorescence staining of the pan-macrophage marker F4/80-positive (green) with the M2 macrophage marker Arginase I-negative (Arg1, red) from KP orthotopic PDAC mouse model sections treated with placebo (n=9), tiplaxtinin (n=5), anti-PD1 mAb and gemcitabine (n=8) or anti-PD1 mAb, gemcitabine and tiplaxtinin (n=9). Gem: gemcitabine. Tip: tiplaxtinin. This panel is related to main Fig. 6I, 6J.

Error bars represent mean ± SD, except (C) that represents mean ± SEM. Statistical analysis was performed using one-way ANOVA. *$p<0.05$, **$p<0.01$, ***$p<0.001$.
